# Atypical presentation of familial hypomagnesemia with hypercalciuria and nephrocalcinosis in a patient with a new claudin-16 gene mutation 

**DOI:** 10.5414/CNCS109595

**Published:** 2019-05-16

**Authors:** Júlia Guasti P. Vianna, Thiago Gabriel Simor, Pamella Senna, Michell Roncete De Bortoli, Everlayny Fiorot Costalonga, Antonio Carlos Seguro, Weverton Machado Luchi

**Affiliations:** 1 Internal Medicine at Cassiano Antonio Moraes Hospital, Federal University of Espírito Santo,; 2 Federal University of Espírito Santo,; 3 Department of Internal Medicine, Division of Endocrinology, Federal University of Espírito Santo, Vitória, ES,; 4 Department of Nephrology, School of Medicine, University of São Paulo, São Paulo, and; 5 Department of Internal Medicine, Division of Nephrology, Federal University of Espírito Santo, Vitória, ES, Brazil

**Keywords:** CLDN16, claudin-16, hypercalciuria, hypomagnesemia, nephrocalcinosis

## Abstract

Familial hypomagnesemia with hypercalciuria and nephrocalcinosis (FHHNC) is an autosomal recessive tubular disorder caused by mutations in genes that encode renal tight junction proteins claudin-16 or claudin-19, which are responsible for magnesium and calcium paracellular reabsorption in the thick ascending limb of Henle’s loop. Progressive renal failure is frequently present, and most of the patients require renal replacement therapy still during adolescence. In this case report, we describe a new homozygous missense mutation on *CLDN16* gene (c.592G>C, Gly198Arg) in a 24-year-old male patient diagnosed with FHHNC after clinical investigation due to incidental detection of altered routine laboratorial tests, who was firstly misdiagnosed with primary hyperparathyroidism. In addition, it illustrates an atypical presentation of this disease, with late onset of chronic kidney disease, improving the phenotype-genotype knowledge of patients with FHHNC.

## Introduction 

Familial hypomagnesemia with hypercalciuria and nephrocalcinosis (FHHNC) is a rare autosomal recessive renal tubular disorder, with unknown prevalence, caused by mutations on *CLDN16* (chromosome 3q28) or *CLDN19* (chromosome 1p34.1) genes firstly described by Michelis et al. [1] in 1972 (Michelis-Castrillo syndrome). These genes codify the tight junction proteins claudin-16 and -19, respectively, responsible for the regulation of the paracellular reabsorption of magnesium (Mg^2+^) and calcium (Ca^2+^) at the thick ascending limb of Henle’s loop. FHHNC is characterized by massive urinary loss of Mg^2+^ and Ca^2+^, with consequent hypercalciuria, nephrocalcinosis (NC), hypomagnesemia, and progressive chronic kidney disease (CKD), which usually lead to end-stage renal disease (ESRD) during childhood or adolescence [2]. Additional manifestations include nephrolithiasis, hypocitraturia, urinary tract infection, hyperuricemia, and secondary hyperparathyroidism. The age of onset and the severity of progression of the disease are heterogeneous and correlated with the genotype [3]. Here we present an unusual phenotype presentation for a novel mutation affecting *CLDN16*. 

## Case report 

A 20-year-old Brazilian man was referred for medical investigation after the incidental finding of increased serum creatinine level (Cr: 1.67 mg/dL). Clinical examination was unremarkable, and although abdominal ultrasound (US) disclosed bilateral NC and nephrolithiasis, the patient was asymptomatic. As shown in Table 1, the main initial laboratorial findings included: high serum parathormone levels (PTH: 227 pg/mL), normal serum calcium and phosphorus levels, and hypercalciuria (Ca_U_: 315 mg/24h). Cervical US depicted an increased size of right inferior parathyroid gland (1.2 cm); however, parathyroid scintigraphy did not show alterations. Despite the normal serum Ca^2+^ levels, it was inferred by the endocrinology service as a primary hyperparathyroidism (PHPT), and the patient underwent partial parathyroidectomy. After the procedure, he maintained high serum PTH levels (374 pg/mL) and progressive renal impairment. 

He was referred to the University Hospital Nephrology Service, where additional investigation revealed (Table 1): hypomagnesemia (Mg^2+^: 1.3 mg/dL), hypermagnesuria (Mg^2+^ excretion fraction – FEMg^2+^ of 15.9%), hypercalciuria, hypocitraturia, hyperuricemia with hypouricosuria, and proteinuria. The Cr was already 2.25 mg/dL, with a glomerular filtration rate (GFR) of 40 mL/min/1.73m^2^ according to CKD-EPI equation. Uroculture was persistently positive with *Escherichia coli,* but the patient reported no urinary symptoms. Besides, the patient underwent ophthalmologic evaluation that evidenced myopia and strabism. Such abnormalities, added to the history of consanguineous parents (first-degree cousins) of German descent, suggested the diagnostic hypothesis of FHHNC. Genetic evaluation for mutations on claudin-16 and -19 genes (*CLDN16* and *CLDN19*, respectively) was performed, and an unpublished mutation on *CLDN16* was identified: c.592G>C (p.Gly198Arg). Interestingly, in laboratory tests of the patient’s family, hypercalciuria was observed in his mother, father, and brother. Hypermagnesuria, hypomagnesemia, and NC/nephrolithiasis were absent in all of them. 

**Figure 1.**
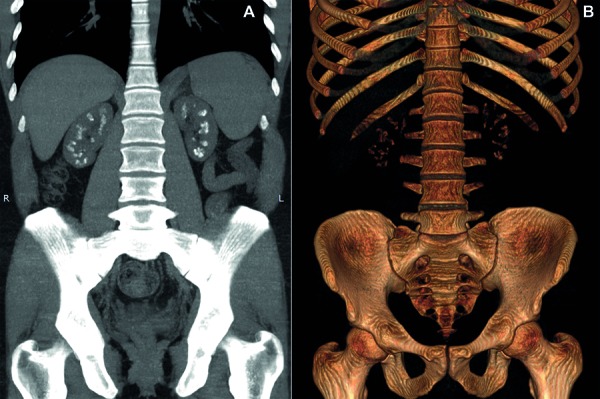
Computed tomography of the abdomen in coronal views demonstrating bilateral medullary nephrocalcinosis. A: coronal average intensity projection; B: 3D reconstruction.

Supportive treatment was implemented with oral magnesium and citrate supplementation (magnesium 100 mEq/day and citrate 100 mEq/day), chlorthalidone 25 mg/day, allopurinol 300 mg/day, and calcitriol 1.25 µg 3 times/week. Renin-angiotensin-aldosterone system (RAAS) inhibitors were not used because of upper-limit potassium levels. There was partial improvement of the serum magnesium level (Mg^2+^: 1.6 mg/dL), reduction of serum PTH (154 pg/mL) and urinary calcium (145 mg/24h). Nevertheless, the patient is presenting progressive decline of renal function, with GFR of 21 mL/min/1.73m^2^ 5 years after the first medical evaluation. During follow-up, nocturia/polyuria was reported and serum sodium levels tended to rise, suggesting a picture of nephrogenic diabetes insipidus. Nephrocalcinosis is illustrated in Figure 1, and evolution of laboratory parameters is shown in Table 1. 

## Discussion 

**Figure 2.**
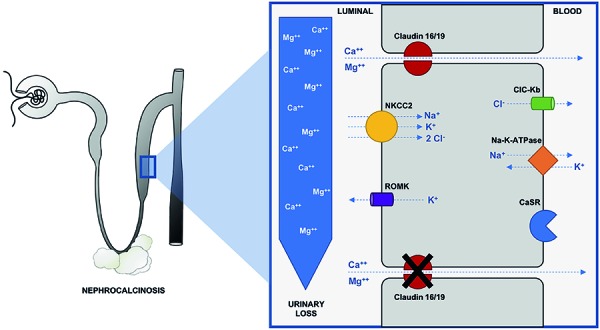
Representation of segmental and cellular distribution of claudin-16 and -19 along the nephron and paracellular reabsorption of Mg^2+^ and Ca^2+^ in the TAL of Henle’s loop. Claudin-16 and -19 are at the tight junctions and they regulate the paracellular diffusion of Mg^2+^ and Ca^2+^. It requires a positive trans-epithelial electrical gradient that is created by ion transporters, including Na-K-ATPase, ClC-Kb (barttin), Ca^2+^-sensing receptor (CaSR), renal outer medullary K^+^ channel (ROMK) and NKCC2. These channels contribute to a lumen-positive voltage that allows Mg^2+^ and Ca^2+^ reabsorption through the claudins. In FHHNC, the loss of claudin function due to mutations or deletions on *CLDN16* or *CLDN19* genes leads to an impaired reabsorption of Mg^2+^ and Ca^2+^, coursing with urinary loss of those ions. It results in elevated FEMg^2+^%, hypomagnesemia, hypercalciuria, and nephrocalcinosis.

Claudins are tight-junction membrane proteins that determine the permeability and selectivity of different ions along the nephron. Claudin-16 (previously known as paracellin-1) and claudin-19 are expressed on the thick ascending limb of Henle’s loop and are responsible for paracellular resorption of ~ 60% of Mg^2+^ and 20 – 30% of Ca^2+^ filtered by the kidneys, driven by transepithelial voltage gradient lumen-positive created in this segment (Figure 2) [2]. Claudin-16 is exclusively expressed in the kidneys, differing from claudin-19, which is also expressed in retina and peripheral neurons [4]. Defects in the genes that encode these proteins result in FHHNC. 

**Figure 3.**
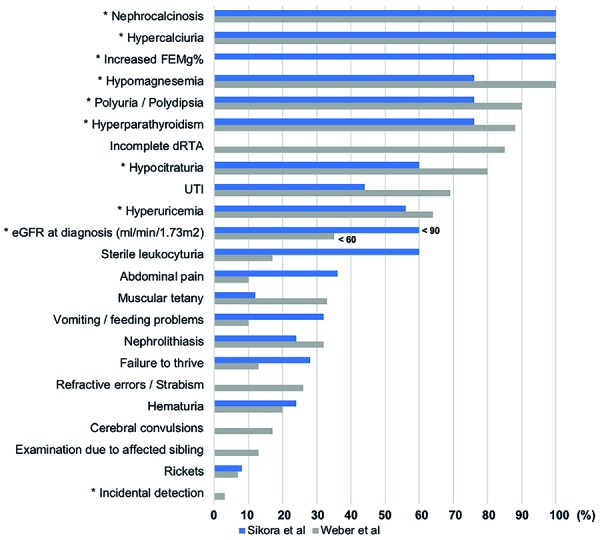
Frequency of occurrence of clinical and biochemical abnormalities in FHHNC according to Weber et al. [1] and Sikora et al. [6]. *Abnormalities that are present in the reported case. Additionally, our patient presents asymptomatic bacteriuria and albuminuria, that were not included in the evaluation (that were not included in the evaluation by these authors). Although urine acidification analysis tests were not performed, the presence of hypocitraturia and urine pH persistently > 5.5 suggests the existence of the dRTA. FEMg^2+^ = fractional excretion of magnesium; dRTA = distal renal tubular acidosis; UTI = urinary tract infection; eGFR = estimated glomerular filtration rate.

This disease is characterized by excessive renal loss of Mg^2+^ and Ca^2+^, leading to hypomagnesemia, hypercalciuria, NC, and progressive CKD. The symptoms generally appear in early childhood or adolescence (mean age of 3.5 – 7.0 years), and most of the patients have significant decrease of GFR at the time of diagnosis; half of them require replacement renal therapy still during adolescence [3, 5]. Figure 2 and Figure 3 illustrate the physiology, variability of initial clinical presentations, and laboratory findings observed during the follow-up period [1, 6]. 

**Figure 4.**
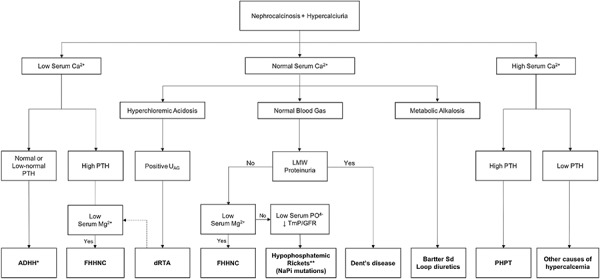
Algorithm for differential diagnosis of nephrocalcinosis associated with hypercalciuria. Ca^2+^ = calcium; U_AG_ = urinary anion gap; dRTA = distal renal tubular acidosis; LMW = low molecular weight; Mg^2+^ = magnesium; FHHNC = familial hypomagnesemia with hypercalciuria and nephrocalcinosis; PTH = parathormone; PHPT = primary hyperparathyroidism; TmP/GFR = renal tubular maximum reabsorption rate of phosphate to glomerular filtration rate; ADHH = autosomal dominant hypocalcemia with hypercalciuria. *Nephrocalcinosis rarely occurs in the initial presentation of ADHH, being observed specially after the treatment of hypocalcemia with calcitriol and calcium. **NaPi mutations may also present with hypercalcemia.

In this case, the incidental detection and late-age onset of CKD are an atypical presentation of FHHNC (Figure 3). The findings of NC and nephrolithiasis and subsequent hypercalciuria, high level of PTH and an increase of the right inferior parathyroid, initially led to a misdiagnosis of PHPT. However, the distinguishing mark of PHPT, which predominantly occurs in postmenopausal women, is the presence of hypercalcemia [7]. As shown in Table 1, the serum calcium levels of the patient did not rise at any time and tended to the lower limit of the reference. Also, the high level of PTH after surgery indicates that the diagnosis of PHPT was inappropriate, suggesting secondary hyperparathyroidism. This was corroborated by the histopathological finding consistent with parathyroid hyperplasia. An algorithmic approach to the evaluation and differential diagnosis of a patient with hypercalciuria and NC is suggested in Figure 4. 

Despite the atypical presentation, the hypothesis of FHHNC was motivated by the following observations: a history of consanguineous parents of German descent, suggesting the possibility of autosomal recessive genetic disease; and a rapid evolution of GFR loss, contrasting with most of the tubular disorders that lead to NC [3]. Thereafter, the marked hypomagnesemia detected with high FEMg (> 4%) was the key point for the diagnosis. However, it is worth mentioning that the absence of hypomagnesemia does not exclude FHHNC from the causes of hypercalciuria-induced NC. Of note, a case study showed that 24% of patients had normal Mg^2+^ serum levels, but 100% of them had increased FEMg^2+^ (mean of 10.6%), reinforcing the importance of the evaluation of FEMg^2+^ [6]. In addition, as CKD advances, Mg^2+^ renal filtration decreases, and hypomagnesemia tends to get milder [2]. 

The persistent and excessive renal calcium loss in FHHNC leads to a tendency toward hypocalcemia that is prevented by the development of secondary hyperparathyroidism. In this scenery, 1.25-dihydroxyvitamin D levels may be initially increased; however, as CKD progresses it reduces, contributing to the elevation of PTH levels [8]. Thus, the serum PTH levels are disproportionately high for the CKD stage, and may rise even before the GFR falls [3]. On the other hand, it contrasts with suppressed PTH serum levels commonly seen in the presence of chronic Mg^2+^ deficiency [6]. 

As described above, another striking feature of FHHNC is the progression to end-stage renal disease (ESRD). In addition to marked medullary NC, and the activation of the inflammasome by crystal nephropathy, this rapid evolution of CKD appears to be related to abnormalities during renal development caused by the claudin mutation itself [2]. A correlation between genotype and the progression of renal failure over time was not established until 2008, when Konrad et al. [3] stratified 71 patients according to their mutation status for genotype/phenotype analysis. This study demonstrated that the presence of at least one allele with partial loss (PL) of function of *CLDN16* (residual function) is associated with a delay in the progression of renal failure. Patients with complete loss (CL) of function in both alleles compared to patients with at least one allele with PL developed faster and more precocious CKD progression. The mean age at onset of symptoms was 2.2 vs. 5.6 years, and mean GFR decrease was 7.3 (5.0 – 9.6) vs. 2.9 (1.6 – 4.9) mL/min/1.73m^2^/year, respectively. In addition, the slope of GFR fall drastically increases as CKD progresses [1]. The loss of GFR > 6.5 mL/min/1.73m^2^/year in the last 5 years of follow-up (GFR from 58 to 21 mL/min/1.73m^2^) suggests that our patient could have CL of function in both alleles, although the incidental onset presentation and the late age at ESRD are unusual in this genotype subgroup. 

More than 50 mutations on *CLDN16* and 17 mutations on *CLDN19* have been described, and most of them are missense mutations. These mutations are found especially in families from Germany, Eastern European countries, France, and Spain [2]. A homozygous mutation not previously described on *CLDN16* was detected in our patient: c.592G>C. This is a missense mutation located in the first base of codon 198, in which guanine is switched for cytosine, resulting in the substitution of the amino acid glycine for arginine (G198R). Two other mutations on codon 198 were previously described but resulted in other amino acids substitutions: a) G198A (guanine switched for cytosine in the second base of the codon, with amino acid glycine being substituted for alanine) [1]; and b) G198D (guanine switched for adenine in the second base of the codon, with amino acid glycine being substituted for aspartic acid) [9]. Interestingly, the pattern of CKD progression in a patient with G198A mutation, who has CL of function in both alleles, resembles our case. We did not find evolution data in a patient with G198D mutation. It is worth mentioning that a similar phenotype was recently described in a patient with late onset of CKD and rapid evolution to ESRD; however, in this case FHHNC was caused by a large deletion in *CLDN16*.[10] 

The urine acidification defect often present in FHHNC is related both to distal renal tubular acidosis (mostly incomplete), that explains hypocitraturia and persistently alkaline urine pH (> 5.5) observed in these cases, and to defective ammonia transfer to the distal nephron [11]. Hyperuricemia in FHHNC could not be completely explained by the GFR deterioration since it is described despite normal renal function. It is speculated that interstitial nephropathy/NC itself could then be related to reduced uric acid excretion and distal acidification defect [6, 11]. Of note, the albuminuria existent in this report has been rarely described in FHHNC patients, suggesting that in some cases the glomerular filtration barrier damage occurs concurrent to CKD progression [12]. Additionally, although severe ocular involvement is a hallmark characteristic of *CLDN19* mutations (macular colobomata, myopia, and horizontal nystagmus), some mild ophthalmologic abnormalities have been described in patients with *CLDN16* mutations, including refractive errors and strabismus as observed in our patient [4]. Besides, mutations in *CLDN19* may also present with neurological manifestations, mainly exercise intolerance, weakness, and cramps. 

There is not a specific treatment for this disease, and the current therapy is basically supportive, consisting in oral Mg^2+^ and citrate supplementation as well as thiazide diuretics to reduce urinary calcium levels and control NC progression [2]. Conversely, thiazide diuretics may worsen hypocitraturia and hyperuricemia, and in our patient, it did not have an impact on calciuria reduction [13]. Renin angiotensin system inhibitors may be used to decrease the rate of progression of CKD [14]. Despite the controversy around the implementation of vitamin D supplementation, because of the risk of increasing intestinal calcium absorption and worsening hypercalciuria, it is expected that it also reduces PTH levels and leads to reduction on hypercalciuria, a fact that occurred in the present case. In addition, it was evidenced that normal vitamin D levels are associated with attenuation of CKD progression [15]. Unfortunately, data from the literature indicate that the treatment does not have considerable effects on progressive renal function loss, and the only definitive treatment is kidney transplantation [2]. However, the late diagnosis of these patients, when CKD is already at an advanced stage, could contribute to the low effectiveness of supportive therapy in reducing the progression rate of CKD. 

Genetic testing is mainly used for the confirmation of the clinical/biochemical/imaging diagnosis, to assess genetic risk in relatives and to guide genetic counselling. Prenatal or pre-implantation genetic testing are feasible for families with known disease-causing variants. Genetic testing establishes an accurate diagnosis of FHHNC even in patients who still have no clinical/laboratorial alterations, allowing an early initiation of the treatment and/or surveillance, in order to slow down disease progression. Genetic evaluation of relatives is also important for the selection of potential family donors for kidney transplantation, and the search for potential donors can be started as soon as FHHNC diagnosis is made. Also, the definition of the mutation on the *CLDN19* gene increases the awareness for the continuous ophthalmological evaluation [16]. 

Although FHHNC is a rare disease, serum and urinary Mg^2+^ analysis and the calculation of FEMg are indispensable for the differential diagnosis of patients presenting with hypercalciuria and NC in the first and second decades of life, especially in those with a family history of consanguinity and rapid GFR decline. This report describes an atypical presentation of a new missense *CLDN16* mutation, contributing to improve the phenotype-genotype knowledge of patients with FHHNC. 

## Funding 

The authors received no financial support for the research, authorship, and/or publication of this article. 

## Conflict of interest 

The authors declared no potential conflicts of interest with respect to the research, authorship, and/or publication of this article. 

**Table 1.** Laboratory data follow-up according to the patient’s age (years).

	Initial (20 years)	After PTX (22 years)	At diagnosis (24 years)	Follow-up (25 years)	Reference values
Blood					
Creatinine (mg/dL)	1.67	2.25	3.2	3.8	0.4 – 1.3
Urea (mg/dL)	39	49	48	87	15 – 45
Sodium (mEq/L)	141	139	147	146	135 – 145
Potassium (mEq/L)	4.3	4.2	5.7	4.2	3.9 – 5.0
Magnesium (mg/dL)	–	–	1.3	1.67	1.8 – 2.5
Calcium (mg/dL)	8.9	8.5	9.2	10.4	8.8 – 11.0
Phosphorus (mg/dL)	2.6	3.35	3.38	3.6	2.5 – 4.8
Bicarbonate (mmol/L)	22.4	–	26.6	–	22 – 26
Intact PTH (pg/mL)	227	374	599	154	12 – 72
25-OH-Vitamin D (ng/mL)	23	28	44	26	30 – 100
1.25-OH-Vitamin D (pg/mL)	–	–	65.3	–	15.2 – 90.1
Uric acid (mg/dL)	8.5	13.4	13.0	8.4	2.5 – 7.0
Albumin (g/dL)	4.3	–	4.4	–	3.5 – 5.2
Glucose (mg/dL)	94	94	101	100	60 – 99
Alkaline phosphatase (u/L)	68	84	59	–	40 – 129
Urine					
24-hour					
Urinary volume (mL)	2,900	2,970	3,020	2,800	< 3,000
Proteinuria (mg/24h)	1,682	2,480	2,861	–	< 150
Albuminuria (mg/24h)	–	–	–	2,765	< 30
RBP (mg/L)	0.3	–	–	–	≤ 0.4
Calcium (mg/24h)	315	–	306	145	< 200
Sodium (mEq/24h)	–	–	127	112	43 – 220
Potassium (mEq/24h)	–	–	39	16	25 – 125
Magnesium (mg/24h)	–	–	112	151	73 – 122
FEMg^2+^ (%)	–	–	15.9	19.4	2 – 4
Phosphorus (mg/24h)	986	–	984	589	340 – 1,000
Citrate (mg/24h)	–	–	39	60	320 – 1,240
Oxalate (mg/24h)	–	–	22	5.6	7 – 44
Uric acid (mg/24h)	142	–	256	97	250 – 750
Spot					
pH	–	6.0	6.0	6.0	4.5 – 8
Glucose	Negative	Negative	Negative	Negative	Negative
Red blood cells (/HPF)	2	6	2 – 4	2	< 5
Leukocyturia (/HPF)	Numerous	25	20	Numerous	< 5
Proteinuria	+	++	++	++	Negative
β_2_-microglobulin (ng/mL)	8.3	–	4.01	–	≤ 300

PTX = parathyroidectomy; FEMg^2+^ = fractional excretion of magnesium; RBP = retinol-binding protein.
